# Bis[1-(2-naphthyl­imino­meth­yl)-2-naphtholato-κ^2^ *N*,*O*]copper(II)

**DOI:** 10.1107/S1600536809030694

**Published:** 2009-08-08

**Authors:** Zhenghua Guo, Lianzhi Li, Chengyuan Wang, Jinghong Li, Tao Xu

**Affiliations:** a School of Chemistry and Chemical Engineering, Liaocheng University, Shandong 252059, People’s Republic of China; b Research Center of Medical Chemistry and Chemical Biology, Chongqing Technology and Business University, Chongqing 400067, People’s Republic of China

## Abstract

In the title complex, [Cu(C_21_H_14_NO)_2_], the Cu^II^ atom, lying on an inversion center, is coordinated by two bidentate 1-(2-naphthyl­imino­meth­yl)-2-naphtholate ligands in a *trans* arrangement, forming a slightly distorted square-planar coordination geometry. The mean planes of two naphthyl systems of the ligand make a dihedral angle of 40.32 (11)°.

## Related literature

For general background to Schiff base complexes, see: Gamovski *et al.* (1993 ▶); Tarafder *et al.* (2002 ▶); Yang *et al.* (2000 ▶). For related structures, see: Unver *et al.* (2003 ▶); Wang *et al.* (2007 ▶).
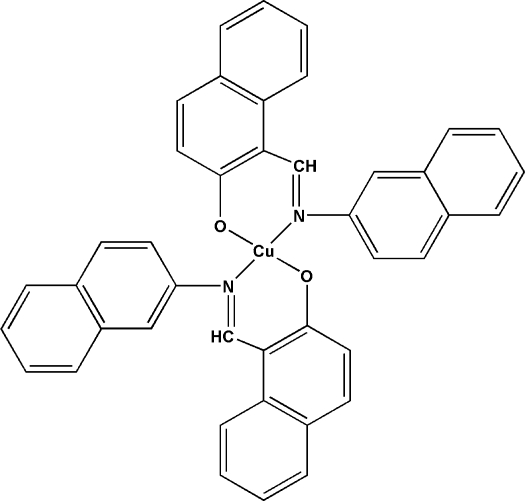


## Experimental

### 

#### Crystal data

[Cu(C_21_H_14_NO)_2_]
*M* *_r_* = 656.20
Monoclinic, 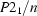

*a* = 5.648 (3) Å
*b* = 18.578 (8) Å
*c* = 14.796 (6) Å
β = 93.635 (5)°
*V* = 1549.4 (12) Å^3^
*Z* = 2
Mo *K*α radiation
μ = 0.75 mm^−1^
*T* = 298 K
0.35 × 0.10 × 0.04 mm

#### Data collection

Bruker SMART 1000 CCD diffractometer
Absorption correction: multi-scan (*SADABS*; Sheldrick, 1996 ▶) *T*_min_ = 0.780, *T*_max_ = 0.971
7695 measured reflections
2721 independent reflections
1869 reflections with *I* > 2σ(*I*)
*R*_int_ = 0.062

#### Refinement

*R*[*F* ^2^ > 2σ(*F* ^2^)] = 0.062
*wR*(*F* ^2^) = 0.121
*S* = 1.10
2721 reflections
214 parameters
H-atom parameters constrained
Δρ_max_ = 0.47 e Å^−3^
Δρ_min_ = −0.75 e Å^−3^

### 

Data collection: *SMART* (Bruker, 2007 ▶); cell refinement: *SAINT* (Bruker, 2007 ▶); data reduction: *SAINT*; program(s) used to solve structure: *SHELXS97* (Sheldrick, 2008 ▶); program(s) used to refine structure: *SHELXL97* (Sheldrick, 2008 ▶); molecular graphics: *SHELXTL* (Sheldrick, 2008 ▶); software used to prepare material for publication: *SHELXTL*.

## Supplementary Material

Crystal structure: contains datablocks global, I. DOI: 10.1107/S1600536809030694/hy2212sup1.cif

Structure factors: contains datablocks I. DOI: 10.1107/S1600536809030694/hy2212Isup2.hkl

Additional supplementary materials:  crystallographic information; 3D view; checkCIF report

## Figures and Tables

**Table 1:** Selected bond lengths (Å)

Cu1—O1	1.874 (3)
Cu1—N1	2.011 (3)

**Fig. 1.**
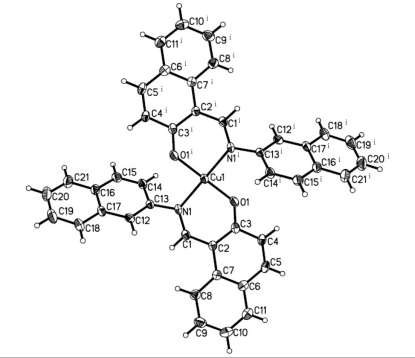
The molecular structure of the title compound. Displacement ellipsoids are drawn at the 30% probability level. [Symmetry code: (i) -x + 1, -y + 1, -z + 1].

**Fig. 2.**
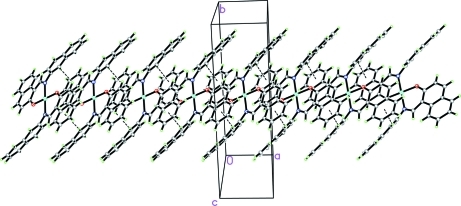
One-dimensional chain structure of the title compound, connected by weak C—H···π interactions (dashed lines).

## supplementary crystallographic information

### Comment

Schiff base complexes play an important role in the stereochemical models of transition metal coordination chemistry, with their easy preparation, diversity and structural variation (Gamovski *et al.*, 1993). They aslo have been intensively investigated owing to their strong coordination capability and diverse biological activities, such as antibacterial, and antitumor activities (Tarafder *et al.*, 2002; Yang *et al.*, 2000). As part of a series of the studies (Wang *et al.*, 2007), we report here the synthesis and structure of the title compound, a new copper(II) complex with a bidentate Schiff base ligand derived from the condensation of 2-hydroxy-1-naphthyldehyde and 2-naphthylamine.

The molecular structure of the title complex is shown in Fig. 1. The Cu^II^ atom, lying on an inversion center, is coordinated by two bidentate ligands in a *trans* arrangement, forming a CuN_2_O_2_ square-planar configuration (Table 1), with the typical values of Cu—O and Cu—N bond lengths (Unver *et al.*, 2003). The mean planes of the chelate ring N1, C1, C2, C3, O1, Cu1 (A), bicycles C2—C11 (B) and C12—C21 (C) make the following dihedral angles: A/B 18.92 (19), A/C 58.14 (12) and B/C 40.32 (11)°. Additionally, the relatively short intermolecular distance H12···C7^i^ (symmetry code: (i) *x* + 1, *y*, *z*) of 2.90Å indicates the possible prescence of C—H···π interaction, which forms a one-dimensional chain structure (Fig. 2).

### Experimental

2-Naphthylamine(0.143 g, 1 mmol) was dissolved in hot methanol (10 ml) and added dropwise to a methanol solution (3 ml) of 2-hydroxy-1-naphthyldehyde (0.172 g, 1 mmol). The mixture was then stirred at 323 K for 2 h. Subsequently, an aqueous solution (2 ml) of cupric acetate hydrate (0.200 g, 1 mmol) was added dropwise and stirred for another 5 h. The solution was held at room temperature for 15 d, whereupon green needle crystals suitable for X-ray diffraction were obtained.

### Refinement

H atoms were positioned geometrically and refined as riding atoms, with C—H = 0.93 Å and with *U*_iso_(H) = 1.2*U*_eq_(C).

### Crystal data

**Table:**

[Cu(C_21_H_14_NO)_2_]	*F*(000) = 678
*M**_r_* = 656.20	*D*_x_ = 1.407 Mg m^−^^3^
Monoclinic, *P*2_1_/*n*	Mo *K*α radiation, λ = 0.71073 Å
Hall symbol: -P 2yn	Cell parameters from 2286 reflections
*a* = 5.648 (3) Å	θ = 2.2–25.2°
*b* = 18.578 (8) Å	µ = 0.75 mm^−^^1^
*c* = 14.796 (6) Å	*T* = 298 K
β = 93.635 (5)°	Needle, green
*V* = 1549.4 (12) Å^3^	0.35 × 0.10 × 0.04 mm
*Z* = 2	

### Data collection

**Table:**

Bruker SMART 1000 CCD diffractometer	2721 independent reflections
Radiation source: fine-focus sealed tube	1869 reflections with *I* > 2σ(*I*)
graphite	*R*_int_ = 0.062
φ and ω scans	θ_max_ = 25.0°, θ_min_ = 1.8°
Absorption correction: multi-scan (*SADABS*; Sheldrick, 1996)	*h* = −6→6
*T*_min_ = 0.780, *T*_max_ = 0.971	*k* = −22→21
7695 measured reflections	*l* = −15→17

### Refinement

**Table:**

Refinement on *F*^2^	Primary atom site location: structure-invariant direct methods
Least-squares matrix: full	Secondary atom site location: difference Fourier map
*R*[*F*^2^ > 2σ(*F*^2^)] = 0.062	Hydrogen site location: inferred from neighbouring sites
*wR*(*F*^2^) = 0.121	H-atom parameters constrained
*S* = 1.10	*w* = 1/[σ^2^(*F*_o_^2^) + (0.*P*)^2^ + 3.3732*P*] where *P* = (*F*_o_^2^ + 2*F*_c_^2^)/3
2721 reflections	(Δ/σ)_max_ < 0.001
214 parameters	Δρ_max_ = 0.47 e Å^−^^3^
0 restraints	Δρ_min_ = −0.75 e Å^−^^3^

### Fractional atomic coordinates and isotropic or equivalent isotropic displacement parameters (Å^2^)

**Table:**

	*x*	*y*	*z*	*U*_iso_*/*U*_eq_	
Cu1	0.5000	0.5000	0.5000	0.0330 (2)	
N1	0.4680 (6)	0.58180 (17)	0.5874 (2)	0.0309 (9)	
O1	0.2210 (5)	0.45773 (16)	0.5372 (2)	0.0411 (8)	
C1	0.3354 (8)	0.5772 (2)	0.6562 (3)	0.0341 (11)	
H1	0.3487	0.6148	0.6977	0.041*	
C2	0.1725 (7)	0.5215 (2)	0.6753 (3)	0.0297 (10)	
C3	0.1107 (8)	0.4686 (2)	0.6098 (3)	0.0340 (10)	
C4	−0.0904 (8)	0.4233 (2)	0.6237 (3)	0.0364 (11)	
H4	−0.1336	0.3884	0.5807	0.044*	
C5	−0.2175 (8)	0.4301 (2)	0.6970 (3)	0.0410 (12)	
H5	−0.3507	0.4013	0.7022	0.049*	
C6	−0.1527 (8)	0.4806 (2)	0.7671 (3)	0.0372 (11)	
C7	0.0473 (8)	0.5251 (2)	0.7577 (3)	0.0327 (10)	
C8	0.1156 (9)	0.5708 (3)	0.8321 (3)	0.0448 (12)	
H8	0.2512	0.5990	0.8298	0.054*	
C9	−0.0143 (10)	0.5740 (3)	0.9069 (3)	0.0552 (14)	
H9	0.0324	0.6050	0.9541	0.066*	
C10	−0.2161 (10)	0.5315 (3)	0.9131 (4)	0.0588 (15)	
H10	−0.3052	0.5348	0.9637	0.071*	
C11	−0.2825 (9)	0.4848 (3)	0.8445 (3)	0.0500 (13)	
H11	−0.4149	0.4556	0.8494	0.060*	
C12	0.7489 (8)	0.6684 (2)	0.6569 (3)	0.0355 (11)	
H12	0.7366	0.6458	0.7125	0.043*	
C13	0.6125 (7)	0.6447 (2)	0.5833 (3)	0.0295 (10)	
C14	0.6260 (9)	0.6813 (2)	0.4998 (3)	0.0398 (11)	
H14	0.5292	0.6669	0.4499	0.048*	
C15	0.7776 (9)	0.7369 (2)	0.4917 (3)	0.0432 (12)	
H15	0.7829	0.7602	0.4362	0.052*	
C16	0.9284 (8)	0.7605 (2)	0.5658 (3)	0.0418 (12)	
C17	0.9088 (8)	0.7268 (2)	0.6506 (3)	0.0362 (11)	
C18	1.0563 (9)	0.7503 (3)	0.7259 (3)	0.0492 (13)	
H18	1.0404	0.7300	0.7827	0.059*	
C19	1.2210 (10)	0.8026 (3)	0.7154 (4)	0.0671 (17)	
H19	1.3191	0.8169	0.7650	0.081*	
C20	1.2455 (10)	0.8353 (3)	0.6308 (5)	0.0677 (17)	
H20	1.3591	0.8709	0.6244	0.081*	
C21	1.1009 (10)	0.8144 (3)	0.5583 (4)	0.0580 (15)	
H21	1.1167	0.8363	0.5025	0.070*	

### Atomic displacement parameters (Å^2^)

**Table:**

	*U*^11^	*U*^22^	*U*^33^	*U*^12^	*U*^13^	*U*^23^
Cu1	0.0312 (4)	0.0303 (4)	0.0371 (4)	−0.0027 (4)	−0.0018 (3)	−0.0056 (4)
N1	0.031 (2)	0.0240 (19)	0.037 (2)	−0.0042 (16)	−0.0053 (18)	−0.0032 (16)
O1	0.0391 (19)	0.0390 (18)	0.0447 (19)	−0.0039 (15)	−0.0004 (16)	−0.0144 (15)
C1	0.030 (3)	0.034 (2)	0.037 (3)	0.001 (2)	−0.005 (2)	−0.005 (2)
C2	0.024 (2)	0.029 (2)	0.035 (2)	0.0015 (18)	−0.0035 (19)	−0.0003 (18)
C3	0.030 (3)	0.031 (2)	0.040 (3)	0.000 (2)	−0.009 (2)	0.001 (2)
C4	0.025 (2)	0.035 (3)	0.049 (3)	−0.003 (2)	−0.005 (2)	0.002 (2)
C5	0.027 (3)	0.041 (3)	0.055 (3)	−0.006 (2)	−0.004 (2)	0.009 (2)
C6	0.035 (3)	0.038 (3)	0.039 (3)	0.008 (2)	−0.001 (2)	0.012 (2)
C7	0.030 (3)	0.032 (2)	0.035 (2)	0.0029 (19)	−0.004 (2)	0.0051 (19)
C8	0.043 (3)	0.054 (3)	0.037 (3)	−0.007 (2)	0.001 (2)	0.005 (2)
C9	0.071 (4)	0.058 (3)	0.036 (3)	0.002 (3)	0.004 (3)	−0.002 (2)
C10	0.062 (4)	0.068 (4)	0.049 (3)	0.005 (3)	0.020 (3)	0.009 (3)
C11	0.045 (3)	0.047 (3)	0.059 (3)	0.000 (2)	0.007 (3)	0.009 (3)
C12	0.043 (3)	0.036 (2)	0.027 (2)	0.003 (2)	−0.003 (2)	−0.002 (2)
C13	0.027 (2)	0.027 (2)	0.033 (3)	0.0024 (19)	−0.001 (2)	−0.0038 (19)
C14	0.047 (3)	0.038 (3)	0.034 (3)	−0.007 (2)	−0.006 (2)	−0.002 (2)
C15	0.054 (3)	0.042 (3)	0.033 (3)	−0.003 (2)	0.000 (2)	0.002 (2)
C16	0.040 (3)	0.034 (3)	0.052 (3)	−0.003 (2)	0.006 (2)	−0.005 (2)
C17	0.030 (3)	0.033 (3)	0.046 (3)	0.001 (2)	−0.003 (2)	−0.012 (2)
C18	0.053 (3)	0.042 (3)	0.050 (3)	−0.002 (3)	−0.016 (3)	−0.009 (2)
C19	0.057 (4)	0.061 (4)	0.081 (5)	−0.011 (3)	−0.013 (3)	−0.027 (3)
C20	0.050 (4)	0.058 (4)	0.094 (5)	−0.018 (3)	0.002 (3)	−0.016 (4)
C21	0.059 (4)	0.045 (3)	0.070 (4)	−0.014 (3)	0.008 (3)	−0.001 (3)

### Geometric parameters (Å, °)

**Table:**

Cu1—O1	1.874 (3)	C9—H9	0.9300
Cu1—O1^i^	1.874 (3)	C10—C11	1.369 (7)
Cu1—N1^i^	2.011 (3)	C10—H10	0.9300
Cu1—N1	2.011 (3)	C11—H11	0.9300
N1—C1	1.304 (5)	C12—C13	1.367 (6)
N1—C13	1.428 (5)	C12—C17	1.417 (6)
O1—C3	1.292 (5)	C12—H12	0.9300
C1—C2	1.424 (6)	C13—C14	1.416 (6)
C1—H1	0.9300	C14—C15	1.352 (6)
C2—C3	1.409 (6)	C14—H14	0.9300
C2—C7	1.449 (6)	C15—C16	1.414 (6)
C3—C4	1.439 (6)	C15—H15	0.9300
C4—C5	1.344 (6)	C16—C21	1.406 (6)
C4—H4	0.9300	C16—C17	1.413 (6)
C5—C6	1.429 (6)	C17—C18	1.418 (6)
C5—H5	0.9300	C18—C19	1.361 (7)
C6—C11	1.401 (6)	C18—H18	0.9300
C6—C7	1.413 (6)	C19—C20	1.406 (8)
C7—C8	1.425 (6)	C19—H19	0.9300
C8—C9	1.367 (7)	C20—C21	1.363 (7)
C8—H8	0.9300	C20—H20	0.9300
C9—C10	1.395 (7)	C21—H21	0.9300
			
O1—Cu1—O1^i^	180.00 (16)	C11—C10—C9	119.7 (5)
O1—Cu1—N1^i^	89.07 (13)	C11—C10—H10	120.1
O1^i^—Cu1—N1^i^	90.93 (13)	C9—C10—H10	120.1
O1—Cu1—N1	90.93 (13)	C10—C11—C6	120.6 (5)
O1^i^—Cu1—N1	89.07 (13)	C10—C11—H11	119.7
N1^i^—Cu1—N1	180.00 (15)	C6—C11—H11	119.7
C1—N1—C13	116.4 (3)	C13—C12—C17	121.6 (4)
C1—N1—Cu1	122.1 (3)	C13—C12—H12	119.2
C13—N1—Cu1	121.0 (3)	C17—C12—H12	119.2
C3—O1—Cu1	129.6 (3)	C12—C13—C14	118.7 (4)
N1—C1—C2	127.8 (4)	C12—C13—N1	121.6 (4)
N1—C1—H1	116.1	C14—C13—N1	119.6 (4)
C2—C1—H1	116.1	C15—C14—C13	121.0 (4)
C3—C2—C1	120.4 (4)	C15—C14—H14	119.5
C3—C2—C7	119.8 (4)	C13—C14—H14	119.5
C1—C2—C7	119.3 (4)	C14—C15—C16	121.4 (4)
O1—C3—C2	124.7 (4)	C14—C15—H15	119.3
O1—C3—C4	117.0 (4)	C16—C15—H15	119.3
C2—C3—C4	118.3 (4)	C21—C16—C17	118.6 (5)
C5—C4—C3	121.8 (4)	C21—C16—C15	123.1 (5)
C5—C4—H4	119.1	C17—C16—C15	118.3 (4)
C3—C4—H4	119.1	C16—C17—C12	119.0 (4)
C4—C5—C6	121.5 (4)	C16—C17—C18	119.1 (4)
C4—C5—H5	119.3	C12—C17—C18	121.9 (4)
C6—C5—H5	119.3	C19—C18—C17	120.2 (5)
C11—C6—C7	120.7 (4)	C19—C18—H18	119.9
C11—C6—C5	120.5 (4)	C17—C18—H18	119.9
C7—C6—C5	118.8 (4)	C18—C19—C20	121.2 (5)
C6—C7—C8	116.8 (4)	C18—C19—H19	119.4
C6—C7—C2	119.5 (4)	C20—C19—H19	119.4
C8—C7—C2	123.7 (4)	C21—C20—C19	119.3 (5)
C9—C8—C7	121.3 (5)	C21—C20—H20	120.4
C9—C8—H8	119.4	C19—C20—H20	120.4
C7—C8—H8	119.4	C20—C21—C16	121.7 (5)
C8—C9—C10	120.8 (5)	C20—C21—H21	119.1
C8—C9—H9	119.6	C16—C21—H21	119.1
C10—C9—H9	119.6		
			
O1—Cu1—N1—C1	19.0 (3)	C2—C7—C8—C9	177.5 (4)
O1^i^—Cu1—N1—C1	−161.0 (3)	C7—C8—C9—C10	1.2 (8)
O1—Cu1—N1—C13	−168.7 (3)	C8—C9—C10—C11	1.3 (8)
O1^i^—Cu1—N1—C13	11.3 (3)	C9—C10—C11—C6	−1.5 (8)
N1^i^—Cu1—O1—C3	161.7 (4)	C7—C6—C11—C10	−0.9 (7)
N1—Cu1—O1—C3	−18.3 (4)	C5—C6—C11—C10	177.9 (4)
C13—N1—C1—C2	178.5 (4)	C17—C12—C13—C14	−2.4 (6)
Cu1—N1—C1—C2	−8.8 (6)	C17—C12—C13—N1	174.0 (4)
N1—C1—C2—C3	−10.4 (7)	C1—N1—C13—C12	47.9 (6)
N1—C1—C2—C7	177.7 (4)	Cu1—N1—C13—C12	−124.9 (4)
Cu1—O1—C3—C2	5.6 (6)	C1—N1—C13—C14	−135.7 (4)
Cu1—O1—C3—C4	−174.8 (3)	Cu1—N1—C13—C14	51.6 (5)
C1—C2—C3—O1	13.0 (6)	C12—C13—C14—C15	2.7 (7)
C7—C2—C3—O1	−175.1 (4)	N1—C13—C14—C15	−173.8 (4)
C1—C2—C3—C4	−166.6 (4)	C13—C14—C15—C16	0.1 (7)
C7—C2—C3—C4	5.3 (6)	C14—C15—C16—C21	175.0 (5)
O1—C3—C4—C5	180.0 (4)	C14—C15—C16—C17	−3.1 (7)
C2—C3—C4—C5	−0.4 (6)	C21—C16—C17—C12	−174.9 (4)
C3—C4—C5—C6	−2.9 (7)	C15—C16—C17—C12	3.3 (7)
C4—C5—C6—C11	−177.5 (4)	C21—C16—C17—C18	2.6 (7)
C4—C5—C6—C7	1.2 (6)	C15—C16—C17—C18	−179.2 (4)
C11—C6—C7—C8	3.2 (6)	C13—C12—C17—C16	−0.6 (7)
C5—C6—C7—C8	−175.5 (4)	C13—C12—C17—C18	−178.0 (4)
C11—C6—C7—C2	−177.6 (4)	C16—C17—C18—C19	−2.8 (7)
C5—C6—C7—C2	3.7 (6)	C12—C17—C18—C19	174.6 (5)
C3—C2—C7—C6	−6.9 (6)	C17—C18—C19—C20	1.4 (8)
C1—C2—C7—C6	165.0 (4)	C18—C19—C20—C21	0.2 (9)
C3—C2—C7—C8	172.2 (4)	C19—C20—C21—C16	−0.4 (9)
C1—C2—C7—C8	−15.9 (6)	C17—C16—C21—C20	−1.1 (8)
C6—C7—C8—C9	−3.4 (7)	C15—C16—C21—C20	−179.2 (5)

Symmetry codes: (i) −*x*+1, −*y*+1, −*z*+1.
